# Outcome of Severe Bacterial Pneumonia in the Era of Pneumococcal Vaccination

**DOI:** 10.3389/fped.2020.576519

**Published:** 2020-12-15

**Authors:** Teresa del Rosal, María Belén Caminoa, Alba González-Guerrero, Iker Falces-Romero, María Pilar Romero-Gómez, Fernando Baquero-Artigao, Talía Sainz, Ana Méndez-Echevarría, Luis Escosa-García, Francisco Javier Aracil, Cristina Calvo

**Affiliations:** ^1^Department of Pediatric Infectious Diseases, Hospital Universitario La Paz and IdiPAZ Research Institute, Madrid, Spain; ^2^Department of Pediatrics, Hospital de Torrejón, Madrid, Spain; ^3^Department of Microbiology, Hospital Universitario La Paz, Madrid, Spain; ^4^Red de Investigación Traslacional en Infectología Pediátrica, Madrid, Spain

**Keywords:** pneumonia, pneumococcal infections, pleural effusion, empyema pleural, *Streptococcus pneumoniae*, *Streptococcus pyogenes*, *Staphylococcus aureus*, pneumococcal vaccines

## Abstract

**Introduction:** After the introduction of pneumococcal conjugate vaccines, community-acquired pneumonia (CAP) caused by *Streptococcus pneumoniae* has decreased whereas *Staphylococcus aureus* and *Streptococcus pyogenes* could be increasing. These bacteria have been associated with high rates of complications.

**Aims:** (1) To describe the characteristics of pediatric bacterial CAP requiring hospitalization. (2) To compare outcomes according to causative microorganisms. (3) To analyze changes in bacterial CAP rate and etiology over time.

**Patients and Methods:** Retrospective single-center study of inpatients aged 1 month-16 years with culture-confirmed bacterial CAP in 2010-2018 in Madrid, Spain.

**Results:** We included 64 cases (42 *S. pneumoniae*, 13 *S. pyogenes* and 9 *S. aureus*). Culture-confirmed CAP represented 1.48-2.33/1,000 all-cause pediatric hospital admissions, and its rate did not vary over time. However, there was a significant decrease in pneumococcal CAP in the last 3 years of the study (78% of CAP in 2010–2015 vs. 48% in 2016-18, *p* = 0.017). Median hospital stay was 10.5 days (interquartile range 5-19.5), 38 patients (59%) developed complications and 28 (44%) were admitted to the intensive care unit. Outcomes were similar among children with *S. pneumoniae* and *S. aureus* CAP, whereas *S. pyogenes* was associated with a higher risk for complications (OR 8 [95%CI 1.1-57.2]) and ICU admission (OR 7.1 [95%CI 1.7-29.1]) compared with pneumococcal CAP.

**Conclusion:** In a setting with high PCV coverage, culture-confirmed bacterial CAP did not decrease over time and there was a relative increase of *S. pyogenes* and *S. aureus*. Children with CAP caused by *S. pyogenes* were more likely to develop complications.

## Introduction

Community-acquired pneumonia (CAP) is one of the most frequent infectious diseases in childhood (1). Pneumonia hospitalization burden is highest among children <5 years old, and ~15% of cases are caused by bacteria (2).

*Streptococcus pneumoniae* is the most common typical bacterial cause of pneumonia in children (2, 3) but *Staphylococcus aureus* and *Streptococcus pyogenes* are becoming increasingly frequent, particularly among children requiring hospitalization and/or presenting with complications such as necrosis, parapneumonic pleural effusion and empyema (4–8). However, few studies have directly compared the clinical characteristics and outcomes of CAP caused by these bacteria according to the etiology.

The introduction of pneumococcal conjugate vaccines (PCV) has led to a substantial reduction of invasive pneumococcal disease and community-acquired pneumococcal pneumonia (9–12). However, their impact in pediatric CAP caused by other bacteria has been barely analyzed (13, 14). The aims of this study were to describe the characteristics of microbiologically confirmed hospitalized bacterial CAP in a setting with high PCV coverage, to compare outcomes according to causative bacteria and to analyze epidemiological changes over time.

## Patients and Methods

A retrospective study of inpatients between the age of 1 month and 16 years with culture-confirmed bacterial CAP during the period 2010–2018 in Hospital La Paz (Madrid, Spain) was performed. The study was approved by the local ethics committee (study number PI-4280).

Cases were identified by electronic medical records search of ICD-10 diagnostic codes for pneumonia, pleural effusion and pyothorax. Patients were included if they had positive blood and/or pleural fluid cultures and met the following definition of CAP: fever with chest radiograph showing focal consolidation with or without pleural effusion, diagnosed by a pediatrician and confirmed by a pediatric radiologist. Complications were defined as significant pleural effusion (>10 mm), empyema, necrotizing pneumonia, severe or impending respiratory failure needing respiratory support (invasive or non-invasive ventilation), and/or signs and symptoms of sepsis or shock. Pneumococcal vaccination was considered as complete in the following scenarios: (a) Patients who started vaccination in the first year of life: at least two doses of PCV at 2-6 months of age, followed by a booster after 11 months; (b) Patients who started vaccination in the second year of life: at least two doses of PCV with a minimum interval of 2 months between them; (c) Patients who started vaccination when they were older than 2 years: 1 dose.

The exclusion criteria were: children who developed pneumonia in the context of suspected bronchoaspiration, those patients who were already admitted to hospital for another reason at the time of diagnosis (nosocomial pneumonia), children with positive blood culture that did not require hospitalization, and blood/pleural fluid isolates that were considered as probable contaminants by the clinical team.

Data were collected anonymously, and included clinical and epidemiological characteristics (age, sex, comorbidities, and vaccination status), laboratory and microbiology results, presence of pleural effusion and short-term outcomes (length of hospital stay, respiratory support, intensive care unit [ICU] admission).

### Microbiological Analysis

Blood and/or pleural-fluid samples were routinely collected for pathogen detection. Blood cultures were incubated in the BD BACTEC FX or BACT/ALERT system at 37°C. If positive, a subculture in conventional media was performed (Columbia agar with 5% sheep blood, Chocolate agar PolyViteX and/or *Brucella* blood agar with hemin and vitamin K1) in aerobiosis/anaerobiosis at 37°C. Pleural fluids were cultured in the same solid media and liquid media (Thioglycolate broth and/or blood culture bottle). Pathogen identification was performed by matrix-assisted laser desorption ionization-time of flight mass spectrometry (MALDI-TOF MS) with optochin test to confirm the identification of *S. pneumoniae*. Antimicrobial susceptibility of the strains was performed by broth microdilution method. *S. pneumoniae* strains were sent to the National Centre for Microbiology (Instituto de Salud Carlos III, Majadahonda) to be serotyped.

### Statistical Analysis

Qualitative data were expressed as absolute and relative frequencies and quantitative data as mean and standard deviation or median and interquartile range (IQR). Categorical variables were compared using chi-square and Fisher's exact test, and continuous variables with Student's *t*-test or non-parametric tests as appropriate. Data were compared according to the microbiological cause of CAP, and causative agents were evaluated in periods of 3 years (2010-2012, 2013-2015, and 2016-2018), in comparison to all-cause hospital admissions in children aged 1 month-16 years. Outcome variables were compared between *S. pneumoniae, S. pyogenes* and *S. aureus* using conditional logistic regression analysis. The relative risks were expressed as odds ratio (OR) with 95% confidence interval (CI). A two-tailed value of *p* < 0.05 was considered statistically significant. All analyses were performed using the Statistical Package for the Social Sciences, version 21.0 (IBM Corp., CA, USA).

## Results

From 2010 to 2018, a total of 66 patients with culture-confirmed CAP were identified. Two of them did not need hospital admission and were excluded (both were older than 3 years and *S. pneumoniae* was isolated in blood). Finally 64 hospitalized children were analyzed (Table 1), 45 of which (70%) were younger than 3 years of age. Twenty-eight patients (44%) were admitted to the pediatric ICU, but in ten cases (36% of ICU admissions) the sole reason was chest drain insertion (6 cases of *S. pneumoniae* CAP and 4 cases of *S. pyogenes*). Almost 60% of children (38/64) developed complications, mainly pleural effusion/empyema (34/64, 53%). Twenty-nine patients with pleural effusion required chest drain insertion (85%). No patient died. Children with complications were older (median age [IQR] 30 [16-59] months vs. 16 [12-31], *p* = 0.015) and had a longer hospital stay (17 [11-27] days vs. 4.5 [1-7], *p* < 0.001).

**Table 1:** Characteristics of patients admitted with culture-confirmed CAP (*n* = 64).

**Gender (males)**	**41 (64%)**
**Median age in months (IQR)**	**21 (13-43)**
**Group of age**	
** <1 year**	**15 (23%)**
**1–3 years**	**30 (47%)**
**>3 years**	**19 (30%)**
Underlying conditions^*^	**16 (25%)**
**Congenital heart disease**	**5 (8%)**
**Other congenital malformations/chromosopathy**	**4 (6%)**
**Asthma**	**3 (5%)**
**Prematurity**	**2 (3%)**
**Other^†^**	**3 (5%)**
**Period:**	
**2010–2012**	**20 (31%)**
**2013–2015**	**17 (27%)**
**2016–2018**	**27 (42%)**
**Etiology**	
***S. pneumoniae***	**42 (66%)**
***S. pyogenes***	**13 (20%)**
***S. aureus***	**9 (14%)**
**Viral coinfection**	**14 (22%)**
**Blood leukocytes/mm^3^ (SD)^‡^**	**19,380 (9302)**
**C-reactive protein mg/L (SD)^‡^**	**168 (137)**
**Duration of stay (median days, IQR)**	**10.5 (5–19.5)**
**Complications^§^**	**38 (59%)**
**Significant pleural effusion/empyema**	**34 (53%)**
**Necrotizing pneumonia**	**8 (13%)**
**Sepsis/shock**	**10 (16%)**
**Admission to PICU**	**28 (44%)**
**Respiratory support^¶^**	**19 (30%)**
**Non-invasive ventilation**	**18 (28%)**
**Mechanical ventilation**	**2 (3%)**

*IQR, interquartile range; SD, standard deviation; PICU, pediatric intensive care unit*.

* *One patient had Down's syndrome and atrial septal defect*.

† *Acute lymphoblastic leukemia, Dravet syndrome, cerebral palsy*.

‡ *At admission*.

§ *Some patients developed more than one complication*.

¶ *One patient required both non-invasive and invasive ventilation*.

*S. pneumoniae* was the most frequently isolated bacteria, accounting for two thirds of the cases (42/64), followed by *S. pyogenes* and *S. aureus*. Bacteria were isolated in blood culture in 35 cases (54%), pleural fluid in 26 (41%) and both in 3 (5%). Antimicrobial susceptibility data were available in 36 (86%) *S. pneumoniae* isolates, 13 *S. pyogenes* (100%), and 8 *S. aureus* (89%). There were five *S. pneumoniae* strains resistant to penicillin (14%), and seven resistant to macrolides and clindamycin (17%), but all were susceptible to third-generation cephalosporins. All *S. pyogenes* were susceptible to penicillin, macrolides and clindamycin. As for *S. aureus*, two were resistant to clindamycin (25%), one was cotrimoxazole-resistant and other methicillin-resistant (MRSA), 13% each. A viral coinfection was detected in 14 patients (22%), mainly with respiratory syncytial virus (*n* = 4, 29% of those with viral coinfection), influenza (*n* = 2, 14%), and human metapneumovirus (*n* = 2, 14%).

Pneumococcal serotypes were identified in 21 of 28 (75%) positive blood cultures, being 4 of them (19%) vaccine serotypes (serotype 1 in three patients, and serotype 3 in one). The non-vaccine serotypes were 24F (5 cases); 12F, 22F, 23B (2 cases each); 8, 15 B, 17F, 23A, 33F, and 38 (one case each). Globally, pneumococcal vaccination status was known in 41/42 cases of pneumococcal CAP (98%), being 26 (63%) fully vaccinated. Six patients (15%) had not received any dose, four (10%) a single dose and five (12%) had received 2–3 doses before the age of 6 months but no booster as all of them were admitted before 1 year of age. Regarding the four children with PCV-13 serotypes, two were fully vaccinated (both patients had CAP caused by serotype 1), one had received only one dose of PCV and the other was unvaccinated.

When analyzing the different study periods, culture-confirmed CAP represented 1.48-2.33/1,000 all-cause hospital admissions for children aged 1 month-16 years, and its rate did not vary over time (Table 2, χ2 for linear trend for incidence 0.52, *p* = 0.4706). In the last period of the study, we observed a relative change in pathogens, with a relative reduction of *S. pneumoniae* and a relative increase in non-pneumococcal bacterial CAP (*p* = 0.017). Patients were compared according to CAP etiology (Table 3). There were no significant differences regarding age or underlying diseases, but the rate of complications, ICU admission and need of respiratory support was different among the three bacteria. When pneumococcal pneumonia was compared with *S. pyogenes* CAP, the latter group was found to be at higher risk for complications (OR 8 [95%CI 1.1-57.2]), pleural effusion (OR 9.2 [95%CI 1.3-65.7]), sepsis/shock (OR 4.4 [95%CI 1.9-10]), and ICU admission (OR 7.1 [95%CI 1.7-29.1]). The comparison between *S. pyogenes* and *S. aureus* showed a lower risk for complications (OR 0.36 [95%CI 0.16-0.86], pleural effusion (OR 0.3 [95%CI 0.11-0.75] and ICU admission (OR 0.37 [95%CI 0.14-0.97]) for *S. aureus*. There were no differences regarding the outcomes of S. *pneumoniae* and *S. aureus* CAP.

**Table 2:** Distribution of CAP etiology over time.

**Time period**	**All-cause pediatric hospital admissions**	**Culture-confirmed CAP**	**Disease rate/1,000 hospital admissions**	***S. pneumoniae***	***S. pyogenes***	***S. aureus***
2010–12	10,668	20	1.88	16 (80%)	3 (15%)	1 (5%)
2013–15	11,478	17	1.48	13 (76%)	2 (12%)	2 (12%)
2016–18	11,627	27	2.33	13 (48%)	8 (30%)	6 (22%)

*CAP, community-acquired pneumonia*.

**Table 3:** Comparison of patients according to CAP etiology (*n* = 64).

	***S. pneumoniae***	***S. pyogenes***	***S. aureus***	***p***
	***n* = 42**	***n* = 13**	***n* = 9**	
Median age in months (IQR)	21 (13–48)	21 (18–41)	13 (3–33)	0.323
Group of age				0.472
<1 year	9 (21.4%)	2 (15.3%)	4 (44.4%)	
1-3 years	19 (45.2%)	7 (53.8%)	4 (44.4%)	
>3 years	14 (33.3%)	4 (30.8%)	1 (11.1%)	
Comorbidities	11 (26%)	1 (8%)	4 (44%)	0.188
Viral coinfection	**2 (5%)**	6 (46%)	6 (67%)	** <0.001**
Blood leukocytes/mm^3^ (SD)^*^	20,322 (8,589)	20,238 (11,490)	13,071 (6,586)	0.159
C-reactive protein mg/L (SD)^*^	185 (153)	155 (77)	116 (129)	0.375
Bacteremia	28 (66.7%)	**1 (7.7%)**	7 (77%)	***p*** **<** **0.001**
Median duration of stay (days, IQR)	7 (2–20)	14 (11–25)	11 (9–14)	0.06
Complications^†^	21 (50%)	**12 (92%)**	5 (56%)	***p*** **=** **0.02**
Pleural effusion	18 (45%)	**12 (92%)**	4 (44%)	***p*** **=** **0.006**
Necrotizing pneumonia	6 (14%)	2 (15%)	0	*p* = 0.43
Sepsis/shock	3 (7%)	**6 (46%)**	1 (11%)	***p*** **=** **0.003**
PICU admission	13 (31%)	**11 (85%)**	4 (44%)	*p* = 0.003
Respiratory support	**5 (12%)**	8 (61.5%)	6 (67%)	***p*** **<** **0.001**
Pleural drainage	13 (31%)	**12 (92%)**	4 (44%)	***p*** **<** **0.001**

*Groups with significant differences appear in bold*.

*IQR, interquartile range; SD, standard deviation; PICU, pediatric intensive care unit*.

* *At admission*.

† *Some patients developed more than one complication*.

## Discussion

In this study conducted in an area of high PCV coverage, we have observed a significant decrease in pneumococcal CAP in the last years of the study, together with an increase of cases caused by *S. pyogenes* and *S. aureus*. Children with *S. pyogenes* CAP were at an increased risk for complications, including sepsis, significant pleural effusion and ICU admission. Clinical outcomes were similar in patients with *S. pneumoniae* and *S. aureus* CAP.

Few data describing non pneumococcal bacterial CAP in children are available. Among those with bacteremic pneumonia, patients infected by *S. aureus* and *S. pyogenes* have been reported to experience increased morbidity and complications compared with children with *S. pneumoniae* (15). In our study, this has been the case for *S. pyogenes*, whereas the outcomes of patients with *S. pneumoniae* and *S. aureus* CAP were similar.

*S. pyogenes* CAP has been previously reported to cause more effusions and morbidity than *S. pneumoniae*, including longer hospital stay and more frequent ICU admission (7). In our series, patients with *S. pyogenes* CAP had the highest rates of pleural effusion and ICU admission. Most studies that have compared children with *S. pneumoniae* and *S. pyogenes* CAP have only included patients with empyema. Among them, *S. pyogenes* is also associated with a more acute and severe presentation (4, 6). These findings are important as invasive *S. pyogenes* infections are rising in the last decades worldwide (16–19), and pediatric parapneumonic pleural effusion in increasingly caused by *S. pyogenes* (5, 14, 20). Therefore, prompt suspicion of *S. pyogenes* CAP is needed, as treatment adjustment can be required.

Community-associated *S. aureus* pneumonia remains rare in children, and there is little information about its clinical characteristics (8, 21). A recent study from Greece observed an increasing trend in *S. aureus* CAP, but most cases were caused by methicillin-resistant *S. aureus* (8). In the cohort of children enrolled in the Etiology of Pneumonia in the Community (EPIC) study in the US, staphylococcal pneumonia was detected in 23 of 2138 children (1%). When compared with children with non-staphylococcal bacterial pneumonia, those with staphylococcal CAP were more likely to have a parapneumonic effusion, had longer hospital stay and ICU admission was more common. Seventeen out of 23 cases were caused by MRSA (21). These results are different from our series, in which the clinical course of *S. aureus* CAP was similar to pneumococcal pneumonia. One potential explanation is the low rate of MRSA in our study (13%), as MRSA CAP has been associated with more severe clinical outcomes among adults with pneumonia (22).

Prior to the introduction of PCV, *S. pneumoniae* was the most common bacterial etiology of typical CAP among pediatric inpatients (23). The 7-valent PCV was introduced in 2000–2001 leading to a striking decrease in invasive pneumococcal disease, but emerging serotypes were detected soon thereafter (24). In the Madrid area, PCV7 was implemented in October 2006, and replaced by PCV-13 in June 2010. The vaccine was only available for private purchase between May 2012 and March 2015, leading to lower vaccination coverage in that period. However, significant reductions in all forms of invasive pneumococcal disease, including bacteremic pneumonia and empyema, have been observed (25), similar to what has been reported in other countries (26). In our series, more than 80% of pneumococcal isolates corresponded to non-vaccine serotypes, as opposed to what has been reported in a multicentric study from France that focused on children with pleural effusion (14). The most frequent non-vaccine serotype among our patients was 24F (24% of cases with serotype identification). After PCV-13 introduction, non-PCV13 serotypes cause a significant proportion of childhood invasive pneumococcal disease, with important regional differences (27). The emergence of serotype 24F has been reported in different European countries, related to the clonal expansion of the ST162 lineage (28, 29).

The effect of PCV vaccination among other bacterial causes of CAP is not well documented. A recent study from Italy including more than five hundred children with parapneumonic effusion (PPE) reports a significant reduction in PPE in PCV-vaccinated children, without increasing PPE due to other bacteria and without serotype shift (13). Data from this study are not fully comparable to ours, as inclusion criteria were different (only children with PPE), microbiological diagnosis was reached in <50% of patients and most children with *S. pneumoniae* infection were not correctly immunized. In our series, the rate of microbiologically confirmed bacterial CAP did not decrease despite high PCV coverage, and the reduction in cases caused by *S. pneumoniae* was coupled with an increase in non-pneumococcal CAP.

This study has several limitations, including its small sample size, especially in certain subgroups. Patients with culture-confirmed CAP before PCV-7 and PCV-13 introduction were not included in the study, and interrupted time series analysis could not be performed. Due to its retrospective nature, we cannot rule out possible inaccuracies and omissions in the clinical records. It includes only inpatients from a single institution, with culture-confirmed CAP. However, our center is a pediatric tertiary referral hospital with a catchment area of ~80,000 children. We decided to focus on microbiologically-confirmed bacterial CAP to exclude other acute lower respiratory tract diseases that can present with overlapping signs and symptoms and abnormal chest radiograph, such as asthma and viral bronchiolitis (12). We included only culture-confirmed cases as bacterial PCR has only recently been available at our institution for selected cases of culture-negative pleural effusion. It is estimated that up to 35% of pleural fluid cultures are positive (12), whereas molecular testing can perform substantially better, especially for the detection of *S pneumoniae* (30, 31). *S. aureus* and penicillin-resistant pneumococcal isolates are more likely to be positive in culture, which can introduce a bias in epidemiological studies of pleural effusion etiology when only culture methods are used (12). However, as microbiological methods were the same throughout the study period, we believe our results suggest a true relative increase in non-pneumococcal CAP among pediatric inpatients. Our data should be validated in larger multicentric studies using standardized culture-based and molecular methods.

Despite its limitations, we believe that the difference reported in this study in clinical outcomes according to the causative agent of CAP is important. When evaluating children with severe CAP, clinicians should be aware of the potential for non-pneumococcal etiologies that can be associated with increased morbidity, especially *S. pyogenes*. The epidemiological changes observed in our study and other larger cohorts (5, 14) should be considered when choosing empiric antibiotic therapy for children with severe CAP.

## Data Availability Statement

The raw data supporting the conclusions of this article will be made available by the authors, without undue reservation.

## Ethics Statement

The studies involving human participants were reviewed and approved by Hospital Universitario La Paz, Madrid, Spain. Written informed consent from the participants' legal guardian/next of kin was not required to participate in this study in accordance with the national legislation and the institutional requirements.

## Author Contributions

CC conceptualized and designed the study, coordinated and supervised data collection and performed statistical analysis. TR, FB-A, TS, AM-E, LE-G, FA, and CC participated in case clinical management. IF-R and MR-G did microbiological studies. MC, AG-G, IF-R, and MR-G collected data. TR, MC, and AG-G drafted the initial manuscript. All authors reviewed the manuscript for important intellectual content and approved the final version of the report.

## Conflict of Interest

The authors declare that the research was conducted in the absence of any commercial or financial relationships that could be construed as a potential conflict of interest.
